# Supplementary material for: Multi‐dataset identification of innovative feature genes and molecular mechanisms in keratoconus
Source: J Cell Mol Med. 2024 Sep 19;28(18):e70079. doi: 10.1111/jcmm.70079 (PMC11412914; doi:10.1111/jcmm.70079)
Supplement: Supplementary file 2 — Material S2. [file JCMM-28-e70079-s002.zip › 7.GSEA analysis/2.ATOH7/KEGG_SPLICEOSOME.html]

Details for gene set KEGG\_SPLICEOSOME[GSEA]

|  || Dataset | ATOH7.group.cls#h\_versus\_l |
| Phenotype | group.cls#h\_versus\_l |
| Upregulated in class | l |
| GeneSet | KEGG\_SPLICEOSOME |
| Enrichment Score (ES) | -0.16071819 |
| Normalized Enrichment Score (NES) | -0.7055606 |
| Nominal p-value | 0.8768267 |
| FDR q-value | 1.0 |
| FWER p-Value | 1.0 |
Table: GSEA Results Summary

  

Fig 1: Enrichment plot: KEGG\_SPLICEOSOME      
 Profile of the Running ES Score & Positions of GeneSet Members on the Rank Ordered List

  

| SYMBOL | TITLE | RANK IN GENE LIST | RANK METRIC SCORE | RUNNING ES | CORE ENRICHMENT || 1 | SF3B4 | na | 3923 | 0.239 | -0.0735 | No |
| 2 | HSPA6 | na | 5975 | 0.172 | -0.1042 | No |
| 3 | SYF2 | na | 7491 | 0.131 | -0.1261 | No |
| 4 | TCERG1 | na | 8129 | 0.116 | -0.1252 | No |
| 5 | SNRPD3 | na | 9087 | 0.097 | -0.1366 | No |
| 6 | RBMX | na | 9923 | 0.083 | -0.1469 | Yes |
| 7 | SMNDC1 | na | 9996 | 0.081 | -0.1354 | Yes |
| 8 | SNRPC | na | 10063 | 0.080 | -0.1240 | Yes |
| 9 | SLU7 | na | 10640 | 0.071 | -0.1287 | Yes |
| 10 | SNRNP27 | na | 10852 | 0.068 | -0.1234 | Yes |
| 11 | LSM4 | na | 11042 | 0.065 | -0.1180 | Yes |
| 12 | SNRPA1 | na | 11214 | 0.063 | -0.1124 | Yes |
| 13 | SNRPB | na | 11474 | 0.059 | -0.1100 | Yes |
| 14 | WBP11 | na | 11648 | 0.057 | -0.1055 | Yes |
| 15 | PRPF3 | na | 11684 | 0.057 | -0.0971 | Yes |
| 16 | NCBP2 | na | 11822 | 0.055 | -0.0919 | Yes |
| 17 | BCAS2 | na | 11850 | 0.054 | -0.0836 | Yes |
| 18 | DHX15 | na | 11980 | 0.053 | -0.0785 | Yes |
| 19 | PUF60 | na | 12280 | 0.049 | -0.0790 | Yes |
| 20 | PPIE | na | 12293 | 0.049 | -0.0712 | Yes |
| 21 | SNW1 | na | 12302 | 0.049 | -0.0633 | Yes |
| 22 | SNRNP200 | na | 12440 | 0.047 | -0.0594 | Yes |
| 23 | DDX39B | na | 12464 | 0.047 | -0.0523 | Yes |
| 24 | SNRPA | na | 12492 | 0.046 | -0.0454 | Yes |
| 25 | PRPF38A | na | 12510 | 0.046 | -0.0382 | Yes |
| 26 | PRPF31 | na | 12553 | 0.045 | -0.0319 | Yes |
| 27 | SF3A3 | na | 12568 | 0.045 | -0.0248 | Yes |
| 28 | PCBP1 | na | 12620 | 0.045 | -0.0188 | Yes |
| 29 | USP39 | na | 12655 | 0.044 | -0.0124 | Yes |
| 30 | EIF4A3 | na | 12713 | 0.043 | -0.0068 | Yes |
| 31 | LSM2 | na | 12818 | 0.042 | -0.0028 | Yes |
| 32 | SNRPE | na | 12870 | 0.042 | 0.0027 | Yes |
| 33 | PPIL1 | na | 12979 | 0.041 | 0.0064 | Yes |
| 34 | HNRNPC | na | 12982 | 0.041 | 0.0131 | Yes |
| 35 | PRPF38B | na | 12994 | 0.041 | 0.0195 | Yes |
| 36 | LSM6 | na | 13114 | 0.039 | 0.0226 | Yes |
| 37 | DHX16 | na | 13260 | 0.038 | 0.0247 | Yes |
| 38 | SRSF7 | na | 13349 | 0.037 | 0.0283 | Yes |
| 39 | SF3B2 | na | 13397 | 0.036 | 0.0330 | Yes |
| 40 | AQR | na | 13563 | 0.035 | 0.0340 | Yes |
| 41 | SNRPF | na | 13636 | 0.034 | 0.0376 | Yes |
| 42 | CTNNBL1 | na | 13708 | 0.033 | 0.0411 | Yes |
| 43 | PQBP1 | na | 13777 | 0.033 | 0.0446 | Yes |
| 44 | HNRNPM | na | 13880 | 0.032 | 0.0469 | Yes |
| 45 | SRSF4 | na | 13902 | 0.031 | 0.0516 | Yes |
| 46 | XAB2 | na | 13910 | 0.031 | 0.0566 | Yes |
| 47 | SNRPD2 | na | 13933 | 0.031 | 0.0612 | Yes |
| 48 | HSPA1L | na | 14016 | 0.030 | 0.0639 | Yes |
| 49 | PRPF4 | na | 14122 | 0.029 | 0.0657 | Yes |
| 50 | DDX23 | na | 14177 | 0.029 | 0.0690 | Yes |
| 51 | THOC3 | na | 14203 | 0.029 | 0.0730 | Yes |
| 52 | PPIH | na | 14378 | 0.027 | 0.0724 | Yes |
| 53 | SF3A2 | na | 14387 | 0.027 | 0.0767 | Yes |
| 54 | SNRNP40 | na | 14660 | 0.024 | 0.0728 | Yes |
| 55 | TXNL4A | na | 14812 | 0.023 | 0.0722 | Yes |
| 56 | SNRPB2 | na | 14846 | 0.023 | 0.0750 | Yes |
| 57 | SF3B6 | na | 14850 | 0.022 | 0.0787 | Yes |
| 58 | PRPF8 | na | 14860 | 0.022 | 0.0822 | Yes |
| 59 | U2AF2 | na | 15346 | 0.018 | 0.0712 | Yes |
| 60 | PRPF18 | na | 15798 | 0.014 | 0.0605 | Yes |
| 61 | HNRNPK | na | 16046 | 0.013 | 0.0555 | Yes |
| 62 | BUD31 | na | 16064 | 0.012 | 0.0571 | Yes |
| 63 | LSM5 | na | 16122 | 0.012 | 0.0574 | Yes |
| 64 | SNRPD1 | na | 16228 | 0.011 | 0.0562 | Yes |
| 65 | SRSF9 | na | 16269 | 0.011 | 0.0568 | Yes |
| 66 | DHX38 | na | 16304 | 0.010 | 0.0576 | Yes |
| 67 | SNRPG | na | 16332 | 0.010 | 0.0585 | Yes |
| 68 | SF3A1 | na | 16394 | 0.010 | 0.0584 | Yes |
| 69 | SRSF10 | na | 16468 | 0.009 | 0.0577 | Yes |
| 70 | RBM22 | na | 16482 | 0.009 | 0.0588 | Yes |
| 71 | RBM8A | na | 16483 | 0.009 | 0.0603 | Yes |
| 72 | ALYREF | na | 17032 | 0.004 | 0.0451 | Yes |
| 73 | SF3B5 | na | 17071 | 0.004 | 0.0447 | Yes |
| 74 | ZMAT2 | na | 17191 | 0.003 | 0.0417 | Yes |
| 75 | LSM8 | na | 17407 | 0.001 | 0.0356 | Yes |
| 76 | PRPF40B | na | 17429 | 0.001 | 0.0352 | Yes |
| 77 | LSM7 | na | 17533 | -0.000 | 0.0322 | Yes |
| 78 | LSM3 | na | 17611 | -0.001 | 0.0301 | Yes |
| 79 | CHERP | na | 17983 | -0.004 | 0.0200 | Yes |
| 80 | HNRNPA3 | na | 18166 | -0.005 | 0.0157 | Yes |
| 81 | DDX42 | na | 18285 | -0.006 | 0.0133 | Yes |
| 82 | CRNKL1 | na | 18549 | -0.009 | 0.0072 | Yes |
| 83 | HNRNPA1 | na | 18821 | -0.011 | 0.0013 | Yes |
| 84 | SNRNP70 | na | 18836 | -0.012 | 0.0028 | Yes |
| 85 | CCDC12 | na | 18870 | -0.012 | 0.0038 | Yes |
| 86 | PRPF6 | na | 18950 | -0.012 | 0.0036 | Yes |
| 87 | CWC15 | na | 19150 | -0.014 | 0.0002 | Yes |
| 88 | EFTUD2 | na | 19196 | -0.015 | 0.0014 | Yes |
| 89 | RBM17 | na | 19299 | -0.015 | 0.0010 | Yes |
| 90 | DHX8 | na | 19375 | -0.016 | 0.0015 | Yes |
| 91 | MAGOHB | na | 19422 | -0.016 | 0.0029 | Yes |
| 92 | DDX5 | na | 19428 | -0.016 | 0.0055 | Yes |
| 93 | DDX46 | na | 19510 | -0.017 | 0.0060 | Yes |
| 94 | SF3B1 | na | 20026 | -0.022 | -0.0052 | Yes |
| 95 | SF3B3 | na | 20575 | -0.027 | -0.0165 | Yes |
| 96 | MAGOH | na | 20806 | -0.030 | -0.0182 | Yes |
| 97 | ISY1 | na | 20836 | -0.030 | -0.0140 | Yes |
| 98 | HNRNPU | na | 21252 | -0.034 | -0.0203 | Yes |
| 99 | SRSF2 | na | 21362 | -0.036 | -0.0175 | Yes |
| 100 | HSPA1A | na | 21593 | -0.038 | -0.0178 | Yes |
| 101 | HNRNPA1L2 | na | 21700 | -0.039 | -0.0143 | Yes |
| 102 | PLRG1 | na | 21771 | -0.040 | -0.0096 | Yes |
| 103 | U2SURP | na | 21857 | -0.041 | -0.0051 | Yes |
| 104 | HSPA1B | na | 22392 | -0.047 | -0.0127 | Yes |
| 105 | CDC40 | na | 22439 | -0.048 | -0.0061 | Yes |
| 106 | TRA2B | na | 22749 | -0.051 | -0.0064 | Yes |
| 107 | NCBP1 | na | 22794 | -0.052 | 0.0010 | Yes |
| 108 | SRSF3 | na | 22909 | -0.054 | 0.0066 | Yes |
| 109 | SRSF8 | na | 23305 | -0.059 | 0.0050 | Yes |
| 110 | THOC2 | na | 23312 | -0.059 | 0.0147 | Yes |
| 111 | PRPF19 | na | 23681 | -0.065 | 0.0149 | Yes |
| 112 | SART1 | na | 24197 | -0.072 | 0.0121 | Yes |
| 113 | HSPA2 | na | 24524 | -0.077 | 0.0156 | Yes |
| 114 | U2AF1 | na | 24697 | -0.081 | 0.0240 | Yes |
| 115 | SRSF5 | na | 25425 | -0.093 | 0.0185 | Yes |
| 116 | PHF5A | na | 25509 | -0.095 | 0.0319 | Yes |
| 117 | RBM25 | na | 25788 | -0.100 | 0.0405 | Yes |
| 118 | HSPA8 | na | 25938 | -0.103 | 0.0533 | Yes |
| 119 | SRSF6 | na | 26415 | -0.113 | 0.0583 | Yes |
| 120 | SRSF1 | na | 27463 | -0.137 | 0.0510 | Yes |
| 121 | CDC5L | na | 27640 | -0.142 | 0.0695 | Yes |
| 122 | TRA2A | na | 28608 | -0.167 | 0.0694 | Yes |
| 123 | ACIN1 | na | 28770 | -0.172 | 0.0934 | Yes |
| 124 | PRPF40A | na | 29075 | -0.182 | 0.1149 | Yes |
| 125 | THOC1 | na | 31947 | -0.291 | 0.0804 | Yes |
Table: GSEA details [plain text format]

  

Fig 2: KEGG\_SPLICEOSOME      
 Blue-Pink O' Gram in the Space of the Analyzed GeneSet

  

Fig 3: KEGG\_SPLICEOSOME: Random ES distribution      
 Gene set null distribution of ES for **KEGG\_SPLICEOSOME**
